# Seven Tesla MRI in Alzheimer's disease research: State of the art and future directions: A narrative review

**DOI:** 10.3934/Neuroscience.2023030

**Published:** 2023-12-11

**Authors:** Arosh S. Perera Molligoda Arachchige, Anton Kristoffer Garner

**Affiliations:** Faculty of Medicine, Humanitas University, Milan, Italy

**Keywords:** MRI, 7T, ultra-high field, Alzheimer, dementia

## Abstract

Seven tesla magnetic resonance imaging (7T MRI) is known to offer a superior spatial resolution and a signal-to-noise ratio relative to any other non-invasive imaging technique and provides the possibility for neuroimaging researchers to observe disease-related structural changes, which were previously only apparent on post-mortem tissue analyses. Alzheimer's disease is a natural and widely used subject for this technology since the 7T MRI allows for the anticipation of disease progression, the evaluation of secondary prevention measures thought to modify the disease trajectory, and the identification of surrogate markers for treatment outcome. In this editorial, we discuss the various neuroimaging biomarkers for Alzheimer's disease that have been studied using 7T MRI, which include morphological alterations, molecular characterization of cerebral T2*-weighted hypointensities, the evaluation of cerebral microbleeds and microinfarcts, biochemical changes studied with MR spectroscopy, as well as some other approaches. Finally, we discuss the limitations of the 7T MRI regarding imaging Alzheimer's disease and we provide our outlook for the future.

## 1. Introduction

**Figure 1.**
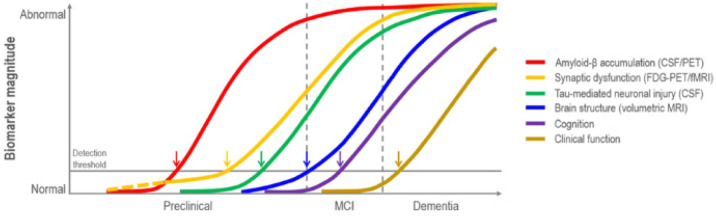
Illustration of the dynamic biomarkers in course of AD pathogenesis which change from normal to maximally abnormal (*y*-axis) as a function of disease stage (*x*-axis). Aβ is identified by cerebrospinal fluid Aβ42 assay or PET amyloid imaging. Synaptic dysfunction evidenced by ^18^F-FDG PET or fMRI, with a dashed yellow line to indicate that synaptic dysfunction may be detectable in carriers of the ε4 allele of the apolipoprotein E gene before detectable Aβ deposition. Neuronal inflammation and injury are evidenced by cerebrospinal fluid tau or phospho-tau, and brain structure is documented by structural MRI.

Alzheimer's disease (AD) is a chronic illness with an estimated incidence of 1–3% and a prevalence of 10–30% in the population >65 years of age. It is the most common cause of dementia and has a high probability of becoming one of the major public health problems in the next 30 years due to the increase in the mean age of the population [1],[2]. AD is characterized by long preclinical and prodromal phases (20 years) and an average clinical duration of 8–10 years. Obtaining a clinical diagnosis remains challenging due to the lack of specific biochemical and morphological changes being recognized early by either non-invasive brain imaging or blood- and cerebrospinal fluid (CSF)-tests. Indeed, differentiating dementia from other causes and common comorbidities is also a challenge [1]. Even though signs of brain atrophy mainly involve the representation of one of the principal biomarkers of neuronal loss visible on magnetic resonance imaging (MRI) within the temporo-parietal cortex and the medial temporal lobe, which may appear too late in the disease course [3]. Fortunately, microscopic autopsy findings have identified several biomarkers based on the pathological hallmarks of AD, namely intracellular neurofibrillary tangles of hyperphosphorylated tau and extracellular beta-amyloid (Aβ) plaques, which could enable diagnosis up to 20 years before the development of clinical symptoms. However, these early pathological changes cannot be directly visualized on conventional MRI [3],[4]. Therefore, structural and functional neuroimaging (e.g., MRI-based volumetric measurements of the hippocampal and entorhinal regions) is used to investigate surrogate biomarkers of AD pathology during its early stages such as mild cognitive impairment (MCI), to obtain an early diagnosis by ruling out other confounding causes of cognitive impairment, and for treatment outcome evaluation, even if it is not currently recommended for a routine diagnostic workup [4],[5]. Figure 1 represents the biomarker evidence-driven model of AD pathogenesis [6].

Neuroimaging using the clinical seven tesla (7T) MRI system is slowly on the rise after the system received approval from the US Food and Drug Administration in 2017 [7]. Here, we discuss the benefits conferred by the utilization of 7T MRI systems, the various neuroimaging biomarkers for AD that have been studied using 7T MRI, and finally, we discuss the technological challenges encountered upon the utilization of the 7T MRI in the context of AD and provide our outlook for the future.

## 2. Technological advances at 7T

**Figure 2.**
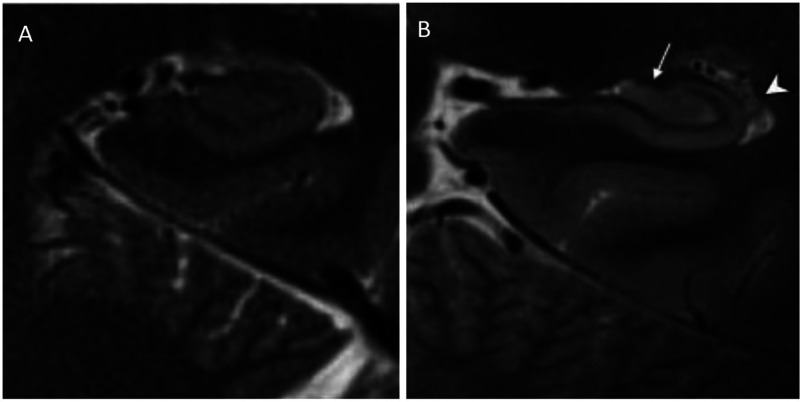
In-vivo hippocampal subfield imaging with high resolution coronal T2 weighted contrast **(A)** at 3T (in-plane resolution: 400 µm) and **(B)** at 7 T (in-plane resolution: 300 µm). Note how alveus (arrowhead), fimbria (white arrow), and stratum layers were resolved at 7 T.

Given the technological advancements seen in the 7T MRI, the main hope of such an expensive technology could be the early identification of AD cases. It is well established that the 7T MRI can provide numerous advantages over conventional field strengths in the non-invasive assessment of normal and pathological anatomy of brain structures involved in AD. Some of the principal factors are a greater contrast-to-noise ratio (CNR), a greater signal-to-noise ratio (SNR), and a submillimeter spatial resolution compared to conventional systems [8]–[11]. Due to these factors, when compared to a 3T system, the enhanced resolution provided by a 7T system facilitates the visualization of the hippocampus and several of its internal features, such as distinguishing the hippocampal mantle from the surrounding anatomical structures [12]. In addition, several studies have shown that advancement from a 3T to a 7T system has allowed for the clearer visualization of micro-anatomic structures including some hippocampal substructures, the layered structure of the neocortex, and the less easily distinguishable lines of Baillarger. See Figure 2 [13],[14]. In recent times, machine learning approaches have been extensively used in attempts to develop algorithms for a reliable early diagnosis of AD. Thus, due to the inadequate quality of frequently employed by 3T MR images, and the typically subpar intensity contrast between the white matter, gray matter, and CSF, they are not the best for supplying accurate ground truth label data for such learning-based training approaches. The dawn of the 7T MRI problem could likely help overcome this problem [15].

At 7T, the temporal resolution of the dynamic phenomena is also increased [16]. This is particularly important in functional MRI (fMRI) studies since it is increasingly being used to identify the temporal dynamics of brain activity. For example, a recent study showed that a higher temporal resolution allows for the optimal functional magnetic resonance imaging (fMRI) blood oxygenation level-dependent (BOLD) sensitivity for high temporal resolution functional network imaging [17]. Furthermore, by using a 7T MRI, one can overcome the low spectral resolution of metabolites during spectroscopic examinations aimed at investigating metabolic changes [18]. Additionally, it has been shown that heavily T2*-weighted imaging sequences at 7T and above have the potential to detect signal alterations given by the parenchymal deposition of paramagnetic substances, like iron and hemosiderin [6]. Indeed, since increased iron and calcium levels have been suggested to be early key AD biomarkers that differentiate AD patients from healthy volunteers, alterations in iron and calcium levels reflect magnetic susceptibility changes. Thus, quantitative susceptibility mapping, which is a useful technique to detect susceptibility changes in cerebral regions with a high accuracy, has been proven reliable in identifying cerebral region abnormalities at early AD stages. In addition, 7T MRI provides a higher sensitivity to susceptibility effects, allowing for the detection of even minute changes in susceptibility, since the sensitivity of MRI to magnetic susceptibility is directly proportional to the strength of the magnetic field. Hence, quantitative susceptibility mapping at 7T MRI may be used to offer information regarding cerebral region abnormalities in early sporadic AD via the direct assessment of biomolecular changes [8],[9],[20].

The primary benefits of higher field strengths for fMRI are the increased nuclear magnetization and susceptibility effects, which result in an increased BOLD contrast. As a result, the higher spatial resolution conferred by the 7T fMRI compared with fMRI at 3T enables the novel insights into brain dysfunction in AD. Hippocampal circuit impairment seen in the earliest stages of AD (that, as a consequence, leads to a decline in explicit memory) is of particular concern when using this technology [21]–[23].

Furthermore, it has been suggested that one of the earliest events in AD is a decrease of cerebral blood flow (CBF). Since the CBF has also been determined through arterial spin labeling (ASL), when the appropriate spin labeling techniques are used, the measurement of CBF at 7T has been shown to benefit from T1 prolongation. ASL was used at submillimetric levels by Kashyap et al. to monitor cortical laminar fMRI responses. This is important because understanding cortical layers will open a new window in elucidating the basis of AD, as the disease is accompanied by specific impairments in laminar-specific circuitry in the brain during its early stages. [8],[24]–[27]. A 7T-optimized technique for vascular wall imaging, such as delay alternating with nutation for tailored excitation prepared imaging, can be utilized to examine vascular wall lesions alongside blood flow. High-resolution ASL perfusion 7T fMRI is an important technique for the in vivo assessment of neurovascular function and metabolic activities of neural circuits across cortical layers, since it allows for quantitative CBF measurements both at rest and during task activation [8],[27],[28].

Finally, it is known that diffusion tensor imaging (DTI) and diffusion kurtosis imaging (DKI) at 7T offer an improved SNR. DKI is an essential imaging technique for the non-invasive measurement of microstructural differences within living tissue. Theoretically, DKI data acquisition at 7T allows for enhanced spatial resolution. DKI can detect changes in brain microstructure between AD patients, MCI patients, and cognitively normal individuals. Additionally, DTI and DKI may serve to compensate for the inability and limitations of traditional MRI to detect pathological changes in microstructure prior to the onset of macroscopic atrophy [29],[30]. Previous research showed that the hippocampal mean kurtosis was the most sensitive single parameter map for discriminating AD patients, MCI patients, and cognitively healthy people [31].

## 3. Brain morphological changes

Until recently, the non-invasive imaging of neuroanatomical atrophy in AD has been limited by a suboptimal resolution. The high spatial resolution obtained by 7T overcomes this limitation as it allows for the improved delineation of hippocampal boundaries (i.e., head, tail, and body) as compared with low-field MRI, even revealing changes occurring during mild cognitive impairment, such as hippocampal subfield atrophy [32],[33]. Indeed, in a recent post-mortem study scanning hemispheres of AD patients and age-matched controls with T2*-weighted 7T MRI, with the aim of evaluating hippocampal subfield atrophy, a significant correlation was demonstrated with the post-mortem analysis of neurofibrillary tangles, amyloid plaques, and neuronal count [34]. Furthermore, it is now well established that AD is not simply an isolated disease with a uniform disease progression. Indeed, since the subtyping of AD is based on the assessment of patterns of brain atrophy on MRI, the higher resolution may provide cues for novel disease patterns [35].

**Figure 3.**
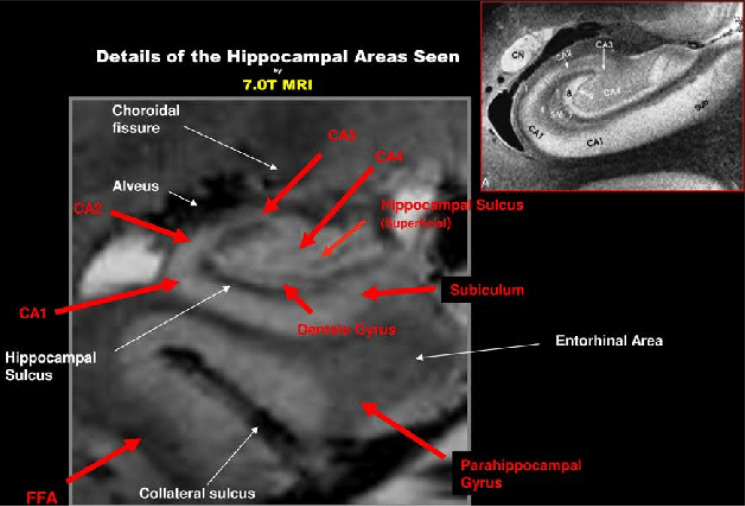
Details of hippocampal areas are clearly visible on 7T MRI. Most of the sub-components of the hippocampal proper near the head of the hippocampus, CA1, CA2 etc. can be clearly seen together with the surrounding parahippocampal cortices such as the subiculum, part of the entorhinal cortex, as well as the fusiform area (FFA).

7T MRI-based segmentation is a promising tool in AD research, thereby allowing us to detect volume loss in distinct hippocampal layers [36]. Numerous 7T studies permitted the analysis of microscopic hippocampal structures and showed that one of the early pathologic changes in AD is a neuronal loss in specific hippocampal subfields. This includes the selective thinning of the CA1 apical neuropil layer in relation to the CA1 cell body layer in subjects with mild AD, and a reduction in the size of the stratum pyramidale of the subiculum, stratum radiatum, stratum lacunosum, stratum moleculare and the dentate gyrus/CA3 as disease severity increases [11],[36]–[38]. Similarly, in a study by Apostolova et al., whose aim was the pathologic validation of the European Alzheimer's Disease Consortium Alzheimer's Disease Neuroimaging Initiative Center Harmonized Hippocampal Segmentation Protocol, they demonstrated significant associations between the hippocampal subfield volume and Braak staging (a method used to classify the severity of neurofibrillary tangle pathology), tau, Aβ burden, and neuronal count. In addition, the hippocampal subfield volume showed significant subfield-wise associations for Aβ in CA1 and the subiculum, tau in CA2, and CA3, and neuronal count in CA1, CA3, and CA4. See Figure 3 [10]. Furthermore, it has been reported that certain 7T MRI sequences, such as the volumic three-dimensional T2 Rapid Acquisition Relaxation Enhancement sequence allowed for the calculation of not only the hippocampal subfield volumes, but even very thin cellular layer volumes, such as the granular cell layer (1 mm^3^) [39]. In addition, several animal model studies on neurodegenerative disease have been conducted using 7T small animal MRI, which has allowed for the assessment of volumes in specific brain structures [40],[41].

As compared to 3T, 7T MRI more than doubles the acquired SNR, thereby increasing the spatial resolution obtained while maintaining acceptable scan times. This allows for the investigation of hippocampal subfield volumes and, as a result, its association with vascular risk factors as, historically, vascular risk factors have been associated with an increased risk of AD, as well as hippocampal volume loss [42]. Indeed, a study by Blom et al. involving older people without dementia revealed that several vascular risk factors were associated with smaller hippocampal subfield volumes, though it failed to provide statistical evidence of the differential effects of each vascular risk factor [43].

Even though the cognitive decline in AD is known to be predominantly related to cholinergic depletion, a systematic review of recent studies by Chen et al. provided evidence of locus coeruleus (LC: a brain stem nucleus responsible for noradrenaline production) degeneration as one of its earliest pathological markers [44]. There is a growing interest in in-vivo imaging of the LC to evaluate the possibility of using it as a biomarker, though this approach has been limited by the poor contrast and spatial resolution of standard MRI methods. Furthermore, in-vivo imaging of the LC is usually performed with a 2D T1-weighted Turbo Spin Echo (TSE) MRI sequence, which suffers from several drawbacks at 3T. To mitigate these limitations, Priovoulos et al. developed a fast, high-resolution, LC imaging technique that consisted of a 7T MT (Magnetization Transfer)-weighted turbo FLASH sequence with higher CNR and SNR, thus permitting a reliable localization of the LC compared to the TSE at both 3T and 7T [45].

A study by van Rooden et al. demonstrated cortical phase shifts in in-vivo studies using transverse 2D T2*-weighted gradient recalled echo (GRE) phase images at 7T. Interestingly, the distribution of these phase changes in the brain followed the known cerebral distribution of amyloid deposition in AD. They concluded that phase measurement on T2*-weighted 7T MRI can be used to diagnose AD with a high specificity (93–100%) but with a relatively lower sensitivity (50–69%), while also enabling the differentiation between early and late AD onset [46],[47]. In light of this, since hypometabolism on [^18^F]-Fluorodeoxyglucose positron emission tomography (PET) has a high sensitivity (88.8%) in the diagnosis of AD during MCI, we think that the novel combined simultaneous 7T PET-MRIs could be used to counteract the low sensitivity while retaining the exceptionally high sensitivity provided by 7T MRI [7],[95],[96]. Furthermore, T2*-weighted 7T MRI allows for the mapping of alterations in the medial temporal lobe cortical lamination. A study by Kenkhuis et al. using T2*-weighted 7T MRI of post-mortem hemispheres of AD patients revealed that the lamination was severely disrupted and showed a correlation with layer-specific changes in myelin architecture [34].

Finally, dilated perivascular spaces (PVS) have shown non-specific associations with AD. Conventionally, the assessment of brain PVSs relies on subjective observations of their size, shape, and number on high-resolution and non-fluid-attenuated T2-weighted MRI images at clinical field strengths (≤3 T) [48]. Since PVS structures in healthy subjects are typically <2 mm, higher field strengths are required to obtain more quantitative information and to provide an improved SNR. In light of this, Cai et al. developed an objective and automatic quantification method at 7T in order to automatically segment the small hyperintense fluid-filling PVS structures based on algorithms for spatial gradients, component connectivity, edge-detection, k-means clustering, etc., which provided a sufficient resolution and SNR for the quantitative measurement of PVS volume densities in deep white matter, which would have been challenging for clinical MRI systems (≤3 T) [48].

## 4. Molecular characterization of cerebral hypointensities on T2*-weighted imaging

The great sensitivity of T2*-weighted sequences to paramagnetic materials, such as hemosiderin and iron, justifies the use of 7T MRI to investigate T2*-weighted hypointensities thought to be associated with amyloid plaque formation, neuroinflammation, and microbleeds. By avoiding ionizing radiation and offering a higher spatial resolution than PET, 7T MRI is a non-invasive repeatable test that may provide new tools for evaluating the alleged effects of AD pathology, specifically the iron-related hypointensity and the subtle morphometric alterations of medial temporal lobe structures [47],[49]–[52].

In a recent study, it was observed that the altered cortical contrast of AD patients on 7T MRI was histologically related to myelin-associated iron content alteration and deposition [34]. In addition to revealing amyloid deposition, phase information from T2*-weighted 7T MRI sequences allows for the distinction between early-onset AD and late-onset AD based on differences in iron deposition within the cerebral cortex [53]. In particular, a study by van Rooden et al. showed that phase differences between the cortex and the subcortical white matter were larger in early-onset AD compared to late-onset AD, suggesting that the iron load increases with the progression of AD [49]. In fact, autopsies in AD patients showed neuronal iron accumulation on T2*-weighted 7T MRI sequences, which was correlated to tau deficiency, amyloid deposition, and neurofibrillary tangles [47]. Moreover, emerging evidence suggests that the 7T MRI is also capable of accurately detecting iron deposition within activated microglia, which might shed light on the role of the immune system in the pathogenesis of AD [54]. Indeed, according to a 7T in vivo study on amyloid plaques, iron-containing activated microglia are a component of amyloid plaques [32].

Prior to the development of 7T MR systems, only either CSF laboratory tests or PET imaging using radiotracers, binding fibrillary Aβ or tau protein, were capable of detecting Aβ and tau pathologies. Among imaging techniques, the gold standard for the in vivo quantification of Aβ load in AD is currently PET using the ^11^C-labeled Pittsburgh Compound-B (^11^C-PiB), which several groups have successfully used to demonstrate amyloid plaque build-up, thus allowing for the visualization of lesion patterns [46]. However, Amyloid PET has some drawbacks though, including a relatively low spatial resolution, the requirement for a radioactive PiB tracer (ionizing radiation), positive findings in amyloid angiopathy (which is known to co-occur with AD but is not diagnostic), and the inability of the vast majority of PET scanners to simultaneously perform functional and anatomical imaging [55],[56]. In addition, fluorinated versions of PiB have been demonstrated to show nonspecific white matter binding. PiB-PET has an in-plane resolution of approximately 2 × 2 mm^2^, which is opposed to the 0.24 × 0.24 mm^2^ resolution of 7T MRI, which facilitates the elevated accuracy and detailed detection of phase changes compared to PiB-PET [49]. This is a context where integrated 7T PET/MR may be a useful solution to overcome the relatively low resolution of PET [7]. Additionally, a study by Schreiner et al. aimed to investigate the regional correlation between PiB (using PiB-PET) and fluid-attenuated inversion recovery (FLAIR) intensity in cognitively normal elderly subjects. A significant correlation between the two was observed mainly within the hippocampus and the brainstem, thus indicating regional Aβ associated tissue edema [57].

## 5. Evaluation of cerebral microbleeds and microinfarcts

**Figure 4.**
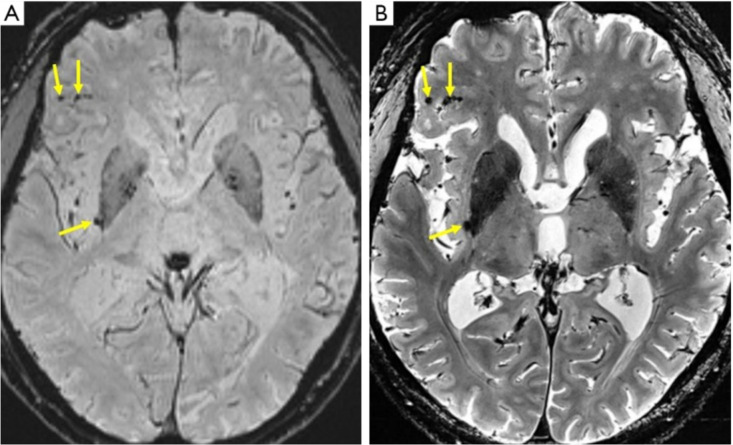
Microbleeds in a 76-year-old patient with AD well visible at both 3T and 7T. Note that the conspicuity of the microbleeds at 7 T is not significantly different from that at 3T. (A) SWI at 3T shows microbleeds as low-intensity spots (arrows), but their relation to the background structure is relatively obscured, and their locations in the cortex or sulci are ambiguous. (B) T2*-weighted image at 7T enables easy detection of microbleeds (arrows), along with precise anatomical localization.

**Figure 5.**
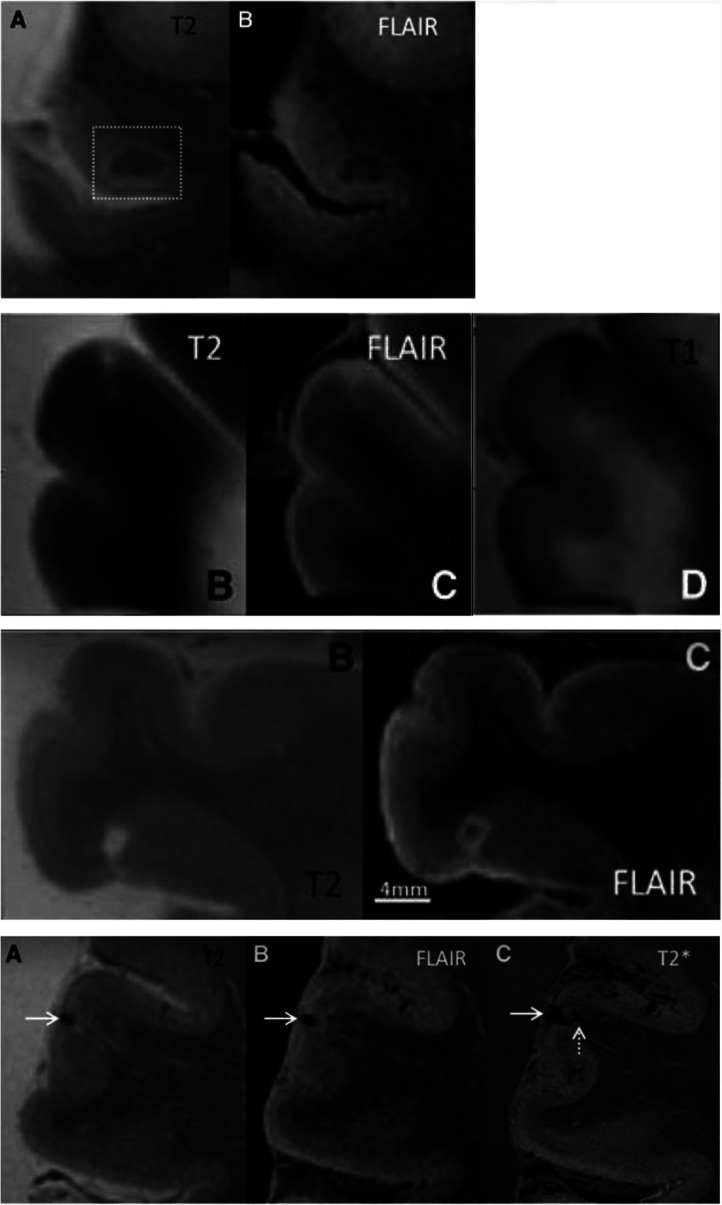
Post-mortem MR images at 7T. **Row 1**: Intracortical acute microinfarct has the same signal intensity as the white matter on T2 (**A**) and FLAIR (**B**). **Row 2**: Intracortical chronic gliotic noncavitated microinfarct appearing hyperintense on T2 (**B**) and FLAIR (**C**), and hypointense on T1 (**D**). **Row 3**: Intracortical chronic gliotic microinfarct with cavitation appearing hyperintense on T2 (**B**), and hypointense surrounded by a hyperintense rim on FLAIR (**C**). **Row 4**: Intracortical chronic gliotic microinfarct with hemorrhagic components (indicated by arrow) appearing hypointense on T2 (**A**) and FLAIR (**B**) and T2∗ (**C**) where it appears enlarged due to the blooming effect. (Courtesy of Dr. Susanne J. van Veluw, Van Veluw Lab for Neuroimaging, Massachusetts General Hospital, Boston, MA, USA).

Apart from morphological changes, in-vivo 7T MRI has allowed for the detection of cortical microinfarcts at increasing frequencies in patients with AD, of which van Rooden et al. demonstrated were particularly associated with cognitive dysfunction [55]. However, it is evident that, in this context and compared with 3T MRI, 7T MRI is of additional value only because it allows for the more precise localization of micro infarcts. Otherwise, as clearly illustrated in Figure 4, the conspicuity of the microinfarct at 7T is similar to that at 3T [8],[55]. Furthermore, van Veluw et al. investigated the spectrum of cortical cerebral microinfarcts (CMI) that could be visualized using 7T postmortem MRI and, based on MR features, classified them into (1) chronic gliotic CMIs—with or without cavitation or hemorrhagic components and (2) acute CMIs. See Figure 5 [58].

A higher prevalence and total number of microbleeds were discovered in patients with either early AD or MCI compared to non-demented subjects, with no observable difference between AD and MCI patients [59],[60]. This was due to the high sensitivity to magnetic susceptibility, which increases the detection rate of microbleeds [61]. Furthermore, compared with susceptibility-weighted imaging (SWI) at 3T, high contrast T2-weighted 7T MRI enables an easier localization of small old hemorrhagic spots within the brain parenchyma [8],[62],[63].

## 6. Metabolic MR imaging studies

**Figure 6.**
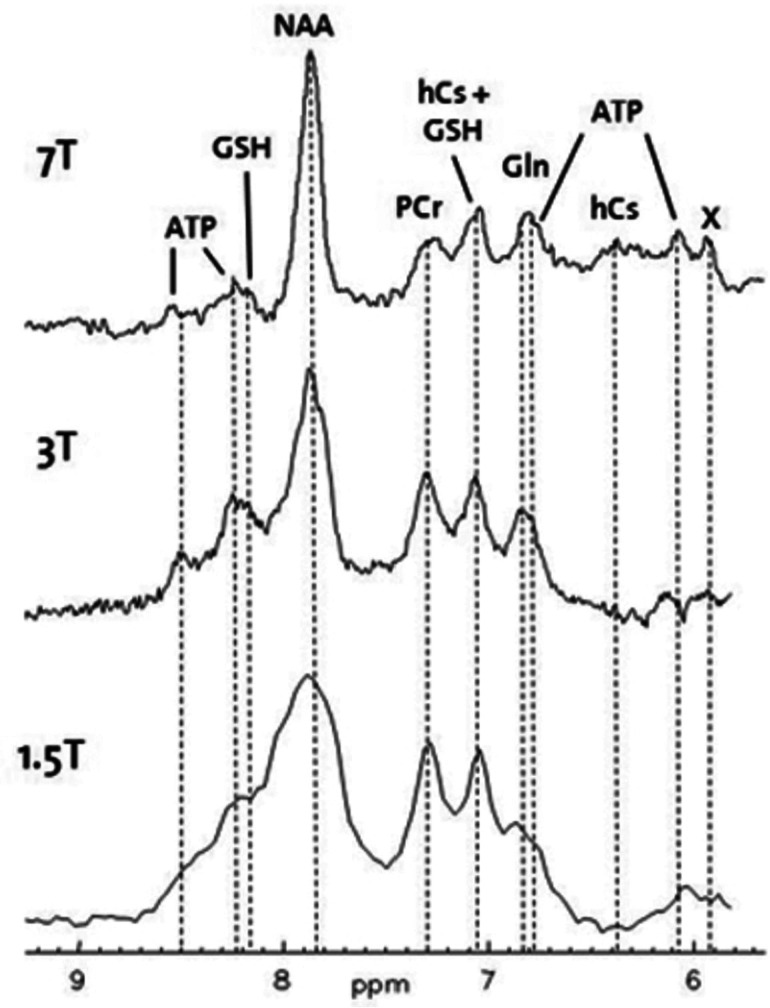
Downfield spectra from human periventricular white matter of the same volunteer at different field strengths. Notice the increase in spectral resolution when with the increase in field strength from 1.5T to 7T. (Courtesy of MR Technology and Methods Group at the Institute for Biomedical Engineering at the University and ETH Zurich).

In addition, higher resolution spectroscopic images and enhanced spectral quantification are produced by combining an increased SNR and an increased spectral quantification of metabolite peaks [64]. The SNR is approximately 1.7 times higher at 7 T relative to 3 T, which also results in a gain in sensitivity, and is particularly prominent for glutamate (Glu), glutamine (Gln), and γ-aminobutyric acid (GABA). See Figure 6 [65]. This is because the relationship between that chemical shift variations and field strength in metabolite resonances is directly proportional [64]. By using 7T MR spectroscopy (MRS), it is possible to measure the signals from 10–15 different metabolites, thereby aiding in understanding the underlying pathophysiological processes [66].

Additionally, the imaging of nonproton nuclei, such as sodium and phosphorus, is made possible by 7T MRI [67]. Technical and methodological advances have made non-invasive measurements of the brain tissue sodium Na^+^ concentration from ^23^Na-MRI more accessible. Compared with traditional ^1^H-MRI, ^23^Na-MRI focuses on detecting sodium alterations as an early sign of neurodegeneration [68]. A study by Haeger et al. discovered elevated cerebral tissue sodium Na^+^ concentrations in 17 biomarker-defined AD patients compared to 22 age-matched control subjects using sodium (^23^Na) magnetic resonance imaging on a 7T MRI scanner [68]. Moreover, it has been recently theorized that mitochondrial dysfunction, which is a neurometabolic hallmark signaling abnormal brain energy metabolism, occurs early in Alzheimer's disease, and that adenosine triphosphate and phosphocreatine are not properly synthesized. With 3T MRI, these molecular levels cannot be accurately measured. However, ^31^P 7T MRS showed that in the temporal lobes of study participants, the ratio of phosphocreatine to adenosine triphosphate, which Das et al. referred to as the energy reserve index, correlated with the participants' cognition levels [69]. Because of the broader availability of 3T MRIs, the technology is evolving in such a way that we may soon be able to modify what we see on 7T scans to be detected with 3T, which will be further facilitated by radiomics analyses and deep learning techniques [69].

Previous studies have suggested a link between neurotransmitter dysregulation and beta-amyloid. A study by Quevenco et al. investigated how Glu and GABA affect the functional connectivity associated with beta-amyloid [70]. For the increased spatial resolution of spectral information and an increased SNR, GABA and Glu were specifically assessed within the gray and white matter of the posterior cingulate and precuneus region using MRS, which was based on the “free induction decay acquisition localized by outer volume suppression” methodology at the ultra-high field (UHF) strength of 7T [70],[71]. GABA and, to a lesser extent, Glu may regulate the functional connectivity associated with beta-amyloid. To fully understand their interplay and potential effects on AD, more research is required [70]. Furthermore, in patients with MCI in AD, recent UHF MRS studies have demonstrated that Glu, which is a key excitatory neurotransmitter in the brain, has been demonstrated to decrease by 5–6%, thereby suggesting that memory impairment in MCI may be caused, at least in part, by disrupted Glu neurotransmission. [72]. Before the advent of UHF systems, it was difficult to accurately detect Glu before the development of UHF scanners due to Glu and Gln's extreme spectrum similarity [73]. On the contrary, the relatively strong combined Glx signal (Gln+Glu) is easily detectable even with standard MRS techniques at lower field strengths [69]. At 7T, discrimination between Glu and Gln is improved, and the Glu estimates can be assumed to be more accurate and specific than at 1.5–3T [69].

Furthermore, chemical exchange saturation transfer (CEST), which is an MRI contrast enhancement technique, enables the indirect and non-invasive determination of the tissue macromolecular content by measuring the exchange of protons between macromolecules and tissue water. A recent study showed that in contrast to wild-type animals, amyloid precursor protein and presenilin-I models of AD and PS19 mice with a genetic propensity for tauopathy exhibit a significant decline in the hippocampal chemical exchange saturation transfer of glutamate (gluCEST) signal with age. In this instance, histology supported the theory that any synaptic loss preceding structural degeneration was related to a decrease in the gluCEST signal [72],[74],[75].

One of the most prevalent metabolites in the human brain, myo-inositol, serves as an osmolyte and is mostly found in glial cells; however, many brain diseases, including AD, are known to change the concentration of MI [76]. According to a study by Haris et al., MI CEST detection in the human brain with 7T MRS provides a high spatial resolution and is possible without going above the permitted limits for the radiofrequency-specific absorption rate [76]. In addition, a study by Marjańska et al. examined how effectively an extended neurochemical profile might be measured noninvasively, with the goal of distinguishing AD from cognitively healthy controls and to better understand the molecular mechanisms behind clinical AD. The levels of 14 neurochemicals in 16 patients with mild to moderate clinical AD and 33 healthy controls were assessed using an ultra-short echo time (8 ms) 7T MRS [77]. The co-occurrence of an increased ascorbate and MI in the posterior cingulate cortex was detected by ultra-short echo time 7T MRS, thus potentially being indicators to separate patients with mild to moderate AD from the controls. In-vivo data from their experiment supported the notion that older persons with clinical AD have peripheral leukocytes in their brains. The central nervous system, which was once thought to be an immune privileged organ lacking a lymphatic system and protected by the blood-brain-barrier (BBB) from the peripheral circulation, is no longer considered as such. Indeed, it has been shown that the BBB can respond to soluble factors and plasma proteins, as well as communicate with peripheral immune system cells to generate neuro-immune system interactions, thereby supporting the idea that neuroinflammation (circulating soluble molecules that mediate immune responses and peripheral immune cells) contributes to the pathophysiology of AD [78]. This factor is important since recent studies have even evaluated the potential use of peripheral leukocytes as biomarkers to diagnose AD [79]. A change from an age-associated pro-inflammatory stage to an AD-associated pathogenic neuroinflammatory response may be marked by elevated myo-inositol and ascorbate concentrations assessed by 7T ultra-short echo time MRS [77]. As a result, their technique may be able to detect the presence of AD before neuronal impairment. Thus, in-vivo 7T MRS might be useful to identify the point at which an intervention might be successful in either stopping or reversing the progression of the disease. Moreover, the use of in vivo 7T MRS to track therapeutic response may be potentially beneficial.

## 7. A glimpse of some other approaches

A 7T-based assessment of subfield-connectivity profiles could provide new insights into some of the existing questions that could not be answered due to the limited resolution of 3T. For example, it is not known if hippocampal dysfunction is more closely related to neurodegeneration than it is to the development of tau- or amyloid pathology (synaptic loss). This is a crucial point since therapies that target tau- or amyloid pathology may be able to only reverse neurodegeneration-independent dysfunction [80]. In addition, pyramidal neuronal intrinsic hyperexcitability, which is already apparent in pre-plaque stages and is accompanied by inhibitory dysfunction, can also be linked to amyloid disease. This inhibitory dysfunction is hypothesized to be the source of network hyperexcitability and hypersynchrony. Despite the fact that fMRI studies are consistent with the presence of pyramidal neuron hyperactivity in preclinical and prodromal AD, it has not yet been determined whether increases in hippocampal activity are due to intrinsic hyperactivity or if it is instead the result of compensatory upregulation of activity in some subfields [81],[82]. Accordingly, it is expected that such 7T-based methods might be useful in assessing the detailed functional connectivity profile of the hippocampus, its subfields, and the perirhinal and subregions of the entorhinal cortices in preclinical AD.

The focus of conventional small-vessel disease neuroimaging markers is on late-stage changes; for this reason, a method of venular assessment at 7T was modified for use in elderly adults as part of a study by Shaaban et al. They observed that venule morphological measurements at 7T may serve as helpful biomarkers for Alzheimer's disease and small-vessel disease. Through future longitudinal studies, apolipoprotein E and vascular endothelial growth factor should be investigated to see how they influence the risk of venular damage [83].

Likewise, Poduslo et al. described various smart molecular probes that, when administered intravenously, enable ex-vivo imaging at the resolution of individual plaques by selectively enhancing them. This could enable early diagnoses and provide a direct measure of the efficacy of anti-amyloid therapies; through future engineering efforts, it could also be used for in-vivo imaging [84]. Their molecular probes targeted amyloid plaques of AD and was detectable by 7T MRI because of contrast imparted by gadolinium labeling. The plaque-to-background tissue CNR, which was precisely correlated with histologically stained plaques, was found to be enhanced more than nine-fold in cortical and hippocampal regions following intravenous administration of these probes in AD transgenic mice [84].

As of recently, MRI at 7T and above, integrated into PET and electroencephalography (EEG) systems, are available for small animal imaging and, hopefully very soon, for clinical systems with larger bores at similar field strengths. Such integrated systems could yield not just superb spatial resolution, but also highly specific molecular data along with temporal mapping [7],[85].

## 8. Limitations of 7T MRI systems

At 7T, the inhomogeneity of the applied radiofrequency (RF) field (B1), inaccuracies in chemical shift localization, and a higher RF power deposition within the patient are among the technical problems. These limit the use of MR spectroscopy, restrict section number/spatial coverage, and produce image artifacts [37]. The RF B1 signal at UHF wavelengths and the dielectric effect lead to an uneven excitation, an inhomogeneous distribution of the signal intensities, a spatial variation in the SNR and CNR, and perhaps even areas of inhomogeneous RF energy deposition in the subject [9],[86],[87]. As RF power consumption is significantly higher and B1 inhomogeneity is more than 2-fold that of 3T systems, absorption rate limits/restrictions are reached much earlier when compared with lower-field scanners [88]. B1+ inhomogeneity can be reduced by using high-permittivity dielectric pads, and signal inhomogeneity correction using parallel RF transmission systems can be used to create perfectly homogeneous images [89],[90].

Intravoxel dephasing due to magnetic susceptibility changes at air-tissue interfaces (e.g., around the mastoids and paranasal sinuses) results in signal losses [91]. On the other hand, this improved contrast, which is based on magnetic susceptibility (T2*-weighted images, phase mapping, SWI, and quantitative susceptibility mapping), is due to an increased sensitivity to the susceptibility properties of tissues at 7T and enables the detection of minute amounts of diamagnetic and paramagnetic (like iron) substances. Its improved sensitivity allows for the visualization of anatomical details and the differentiation of anatomical components with distinct susceptibility properties within brain regions that would otherwise be homogeneous at conventional magnetic field strengths when employed in a “targeted imaging” mode at high resolution [86].

Furthermore, the relaxation times are known to alter when the applied magnetic field is increased, with T1 becoming longer and T2 and T2* becoming shorter. However, although techniques such as arterial spin labeling and time-of-flight angiography can benefit from this, resulting in a quicker acquisition time or better image quality, these relaxation time adjustments are not always beneficial. For example, there is a reduction in gray/white matter contrast compared to conventional field strengths at UHF due to the inconsistency of the prolonged T1 relaxation time between these two regions [9],[87]. Additionally, several authors have acknowledged that the utility of 7T in some patients may be hampered by susceptibility artifacts near significant air-tissue interfaces and the skull base eventually impairs the clear visualization of the hippocampus, especially for fMRI sequences (such as transient spin echo sequences), due to the hippocampus's proximity to the base of the skull [29],[92].

Furthermore, despite the fact that DTI and DKI at 7T can boost the SNR, the inhomogeneity of B0 and B1 and a shorter T2* coupled with rapid signal attenuation resulting from the higher field strength and enhanced susceptibility artifacts have restricted the use of 7T DKI in many ways [30]. Next, the chemical-shift effect, which can enhance the artifacts at the fluid-fat interface, is amplified by an increase in magnetic field strength [9]. Thus, in order to optimize the high-field gain in signal and contrast, minimize the artifacts they produce, deal with the geometrical distortions, there is a need for increased susceptibility artifacts, signal dropouts due to off-resonance frequencies, and patient movement artifacts, customized pulse sequences and innovative hardware solutions [11]. Indeed, the image quality of high resolution T2*-weighted images significantly improved when resonance frequency fluctuations were corrected when using a navigator echo approach. Recently, a method has been developed that can adjust for spatially variable resonance frequency changes, both within and between slices [93]. This method, known as “real-time shimming,” makes use of a calibration scan to connect the motion from the chest wall to the magnetic field's spatial variation. This approach has been effectively used in high resolution T2*-weighted pictures at 7T and during acquisition, where the shim and resonance frequency values were adjusted dynamically [93].

Lastly, some of the practical concerns for 7T MRI are the higher cost of the scanners, siting, and patient experience [37]. Ali et al. described that a quarter of the participants reported induced vertigo while the table was moving at 7 T and approximately 20% had peripheral nerve stimulation [88]. Despite the widespread use of 7T MRI scanners in human clinical research, there are still certain restrictions on the straightforward transfer of ex-vivo outcomes to in-vivo sequences. The need for a lengthy echo time coupled with a long scanning time increases the susceptibility to picture distortions induced by physiological oscillations, patient mobility, or system instabilities [33]. In support of this, several groups have also shown that high quality images could not be achieved in AD patients while utilizing high resolution T2*-weighted sequences at 7T compared with healthy volunteers [94]. The acquisition periods for ex-vivo research are significantly longer than those necessary for clinical protocols; however, the image quality of ex-vivo MRI scans is not compromised by motion artifacts, such as vascular pulsatility or other movement effects. Yet, it is anticipated that advancements in hardware, better acquisition sequences, and post-processing techniques will improve the image quality of clinical 7T MRI and make such ex vivo investigations a more trustworthy benchmark for upcoming clinical trials [33],[97],[98].

## 9. Conclusion and future outlook

Through pathological investigations, significant strides in our understanding of AD have been made. Today's non-invasive technologies, in particular the 7T MRI, can further deepen our understanding of AD pathophysiology, especially in its early phases. Nevertheless, the principal question left is whether the 7T MRI is ready for real-world clinical practice. We have discussed certain instances where the 7T MRI can be useful over the 3T alongside examples where it does not add a significant benefit in the context of AD. Indeed, to prove the value of 7T for disease diagnoses, treatment, and management, more clinical studies are required. For this, one of the major hurdles is the limited availability of 7T MRI systems across the world due to the high costs associated with their installation. Thus, large centres working with patients with AD should consider installing a 7T MRI system if they have the financial means to do so. Additionally, we believe that this process may be facilitated by the introduction of a set of guidelines regarding when imaging at 7T could be recommended, albeit for early screening or for monitoring of disease progression; for this, neuroradiology societies across the globe must take the initiative. Furthermore, the centres with 7T facilities should use it to detect new signs, which can then be re-applied to clinical 3T. The continuous development of high-field imaging and the contemporary advancement in artificial intelligence is expected to result in an enhanced understanding of the pathophysiology and an improved diagnosis and treatment of AD, which will ultimately advance the field.
